# Comparison of clinical efficacy of Ganz approach and K-L approach in treatment of Pipkin type IV femoral head fracture

**DOI:** 10.1186/s12893-025-03301-0

**Published:** 2025-12-05

**Authors:** Jun Zhang, Songqi Chai, Li Dai, Yunqiang Zhuang

**Affiliations:** 1 Department of Trauma Orthopedic Center, Ningbo No.6 Hospital, Ningbo, Zhejiang China; 2 Ningbo Clinical Research Center for Orthopedics, Sports Medicine & Rehabilitation, No.1059 Zhongshan East Road, yinzhou District, Ningbo, 315040 China; 3 Health Science Center, Ningbo University, Ningbo, 315211 China

**Keywords:** Femoral head, Fracture, Fracture internal fixation, Ganz approach, K-L approach

## Abstract

**Objective:**

To compare the efficacy of Ganz approach and Kocher-Langenbeck (K-L) approach in Pipkin type IV femoral head fracture (FHF) with internal fixation.

**Methods:**

The medical records of patients with Pipkin type IV FHF were assessed in the observational study. The patients were divided into Ganz approach group and K-L approach group. The surgical time, intraoperative blood loss, quality of acetabular fracture reduction, Thompson-Epstein clinical and radiological score, fracture healing time, Harris hip score, and complications were recorded.

**Results:**

Fifteen patients were followed for 12–23 months (mean 16.8 months). The Ganz approach group showed significantly better Thompson-Epstein clinical and radiological scores (7 excellent, 1 good) than the K-L group (2 excellent, 5 good) (*P* < 0.05). According to Matta radiographic criteria, anatomical reduction was achieved in 6 cases (with 2 satisfactory) in the Ganz group compared to 5 cases (with 2 satisfactory) in the K-L group, demonstrating a non-significant trend toward superiority for the Ganz approach (*P* > 0.05).

**Conclusion:**

Compared with the traditional K-L approach, the Ganz approach offers better visual field exposure, better reduction quality of FH, and better postoperative functional recovery.

## Introduction

 Pipkin type IV femoral head fracture is defined as a fracture of the femoral head associated with posterior dislocation of the hip joint and an ipsilateral acetabular fracture, which is typically caused by high-energy incidents such as traffic accidents [1]. The typical mechanism of injury for a Pipkin type IV fracture is the transmission of substantial force through the femur to the FH when the hip and knee are flexed, causing the FH to impinge violently on the acetabulum, which leads to fractures of both the FH and acetabulum, usually accompanied by posterior dislocation of the hip.This type of injury is serious and complex, difficult to manage, and has a high complication rate [2]. However, for this kind of fracture, how to minimize the occurrence of FH necrosis, deformity union, malunionsand traumatic hip arthritis, restore the normal anatomic form of the hip, and obtain good clinical function. Open reduction and internal fixation (ORIF) is the gold standard for the treatment of Pipkin fractures[3–5]. The Kocher-Langenbeck (K-L) and Ganz approaches are the main surgical approaches [2, 6, 7]. There remains considerable debate regarding the optimal surgical approach for Pipkin fractures. Some authors recommend the K-L approach, which provides good exposure, allows for repair of posterior wall fractures and the joint capsule, and enables reduction and fixation of femoral head fractures. However, it does not permit direct visualization for the reduction and fixation of fracture fragments located at the anteroinferior aspect of the femoral head. Theoretically, to some extent, the K-L approach may avoid disrupting the anterior capsular blood supply and thus benefit femoral head perfusion [8]. Nevertheless, a comparative study by Stannard et al. demonstrated that the K-L approach further compromises the major posterior blood supply to the femoral head, resulting in an increased incidence of avascular necrosis. In 2001, Ganz et al. [7] described a modified K-L approach, also known as the surgical dislocation technique, for hip joint exposure. This technique allows hip dislocation without disrupting the short external rotator muscles, enabling simultaneous treatment of femoral head fractures and acetabular injuries. Its advantages include providing full exposure of the hip joint while preserving femoral head vascularity, thereby facilitating joint debridement and acetabular fixation [9]. Compared with the traditional K-L approach, the Ganz approach offers superior visualization of the hip joint and anterior acetabular wall; however, it may present technical challenges in the reduction of associated anterior column fractures [10].

However, the efficiency of the two surgeries for Pipkin type IV fractures is unclear.

In the current study, we aimed to assess the clinical effect of K-L and Ganz approaches on the prognosis of FHF (Pipkin type IV fracture).

## Materials and methods

Study design and ethical approval This was a retrospective observational study conducted at Ningbo No.6 Hospital, China, between March 2019 and April 2022. The study was approved by the Medical Ethics Committee of Ningbo No.6 Hospital [Approval NO:2024-15(L)] and conducted in accordance with the Declaration of Helsinki. Written informed consent was obtained from all participants prior to inclusion.

### Inclusion and exclusion criteria

#### Inclusion criteria

Patients aged 23 to 66 years diagnosed with Pipkin type IV fractures were consecutively enrolled from Ningbo No.6 Hospital between March 2019 and April 2022 according to the inclusion and exclusion criteria described below.Inclusion criteria were: (1) According to the diagnostic criteria for Pipkin type IV fracture: FHF combined with ipsilateral acetabular fracture, posterior hip dislocation with failed manual reduction, or posterior hip dislocation with successful manual reduction but a strong desire for surgical treatment; (2) Ganz approach or K-L surgery was used for treatment; and (3) interval between injury and operation < 2 weeks.

#### Exclusion criteria

Exclusion criteria were: (1) previous lower limb dysfunction; and (2) patients with open fracture or severe vascular and nerve injury.

Among them, 8 patients underwent the Ganz approach (Group A), and 7 patients underwent the Kocher-Langenbeck (K-L) approach (Group B).

The allocation of surgical approach (Ganz vs. K–L) was determined preoperatively based on fracture morphology, reducibility, and the surgeon’s preference. Specifically, the Ganz approach was selected for fractures involving the anteroinferior femoral head, comminuted femoral head fragments, or cases requiring circumferential visualization for simultaneous management of acetabular and femoral head injuries. The K–L approach was used when the fracture was predominantly located at the posterior wall of the acetabulum, when closed reduction was satisfactory, or when limited posterior exposure was sufficient for internal fixation. All surgical plans were jointly discussed by two senior orthopedic surgeons before operation to ensure consistency and reduce bias in surgical decision-making.

Surgical methods All patients underwent the following preoperative preparation. Closed reduction was performed in our hospital or in a local hospital in all patients with hip dislocation. After reduction, bone traction was maintained on the tibial tubercle or femoral supracondylar bone, and symptomatic supportive treatment such as anticoagulation and analgesia were given. Pelvic orthostatic and Judet X-ray, pelvic CT plain scan and three-dimensional imaging as well as preoperative examination (blood routine, liver and kidney function, etc.) were completed, and 2–14 days after injury, surgery was performed [11]. Fasting and water incontinence at 8 h preoperatively, a single course of broad-spectrum antibiotics was applied once 30 min before surgery.

Implant selection was determined according to the fracture characteristics: double-head compression screws were applied for larger femoral head fragments, micro-locking plates and screws were used for small posterior wall fractures, and reconstruction plates were used for complex acetabular fractures. Intraoperative decisions, such as the need for trochanteric flip osteotomy or posterior wall fixation, were made based on the stability and quality of fracture reduction under direct visualization. All procedures were performed by two senior orthopedic surgeons with extensive experience in pelvic and acetabular surgery. To maintain consistency, surgical plans were discussed in advance between the two surgeons, and the same operative principles were applied across all cases.

**Fig. 1:**
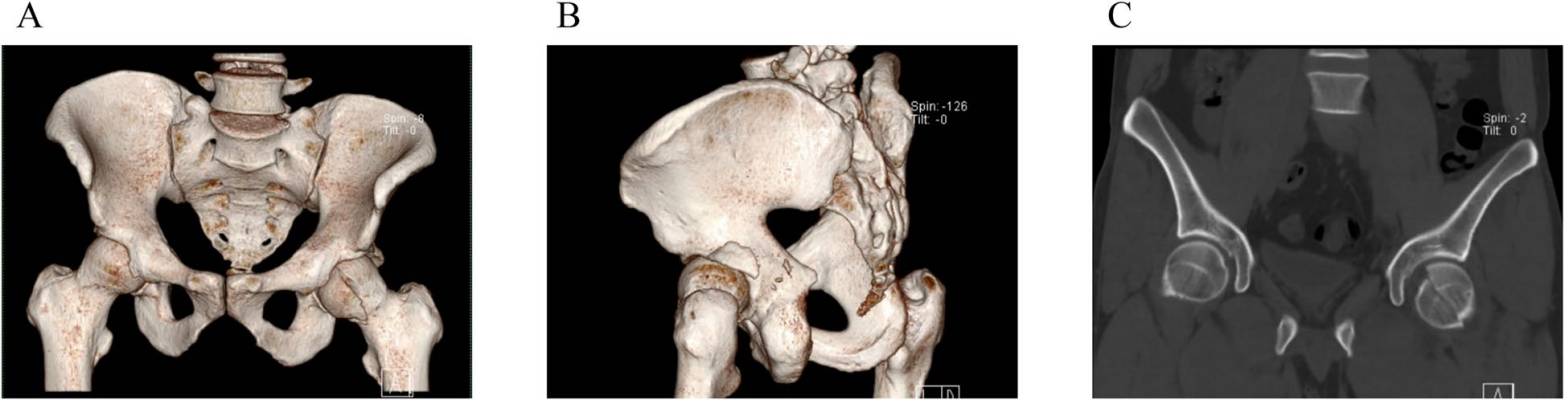
A 32-year-old male patient with a left hip injury caused by a traffic injury Diagnosis: Fracture of the left femoral head (Pipkin type IV), fracture of the left posterior wall of the acetabulum. **A**-**C**: Preoperative CT scan showed a fracture of the left femoral head (Pipkin type IV) with significant displacement of the fracture end and a fracture of the posterior lateral wall of the acetabulum.

**Fig. 2:**
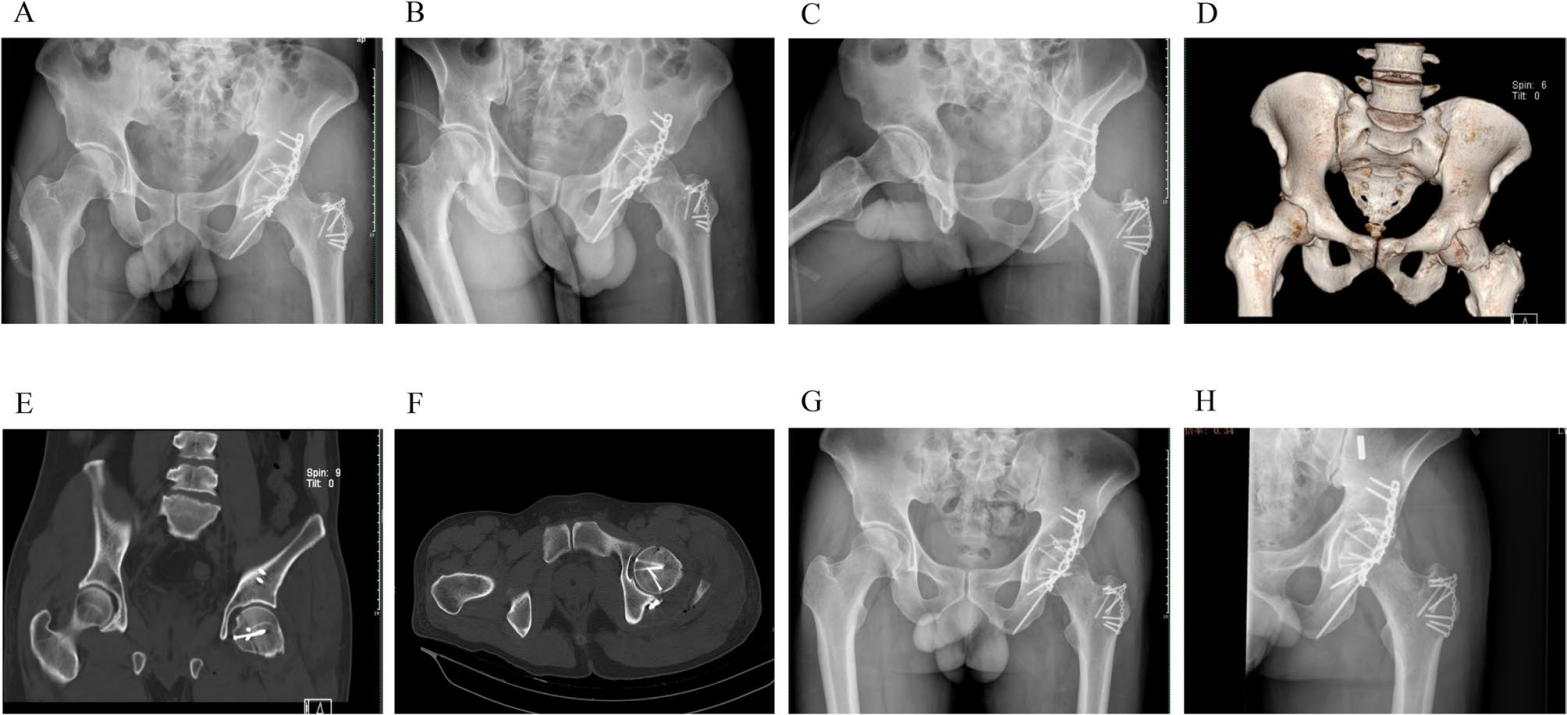
The X-ray and CT images of this male patients **A**-**C**: The X-ray film on the second day after surgery showed that the acetabular fracture was anatomic reduction, and the reduction quality of the femoral head fracture was excellent **D**-**F**: CT scan on the second day after surgery indicated that the acetabular fracture was anatomic reduction, and the reduction quality of femoral head fracture was excellent **G**: Radiographs 1 month after surgery showed that femoral head fractures and acetabular fractures were in good position and internal fixation was firm **H**: Radiographs 3 months after surgery showed that fractures of the femoral head and acetabulum were healed and internal fixation was firm

**Fig. 3:**
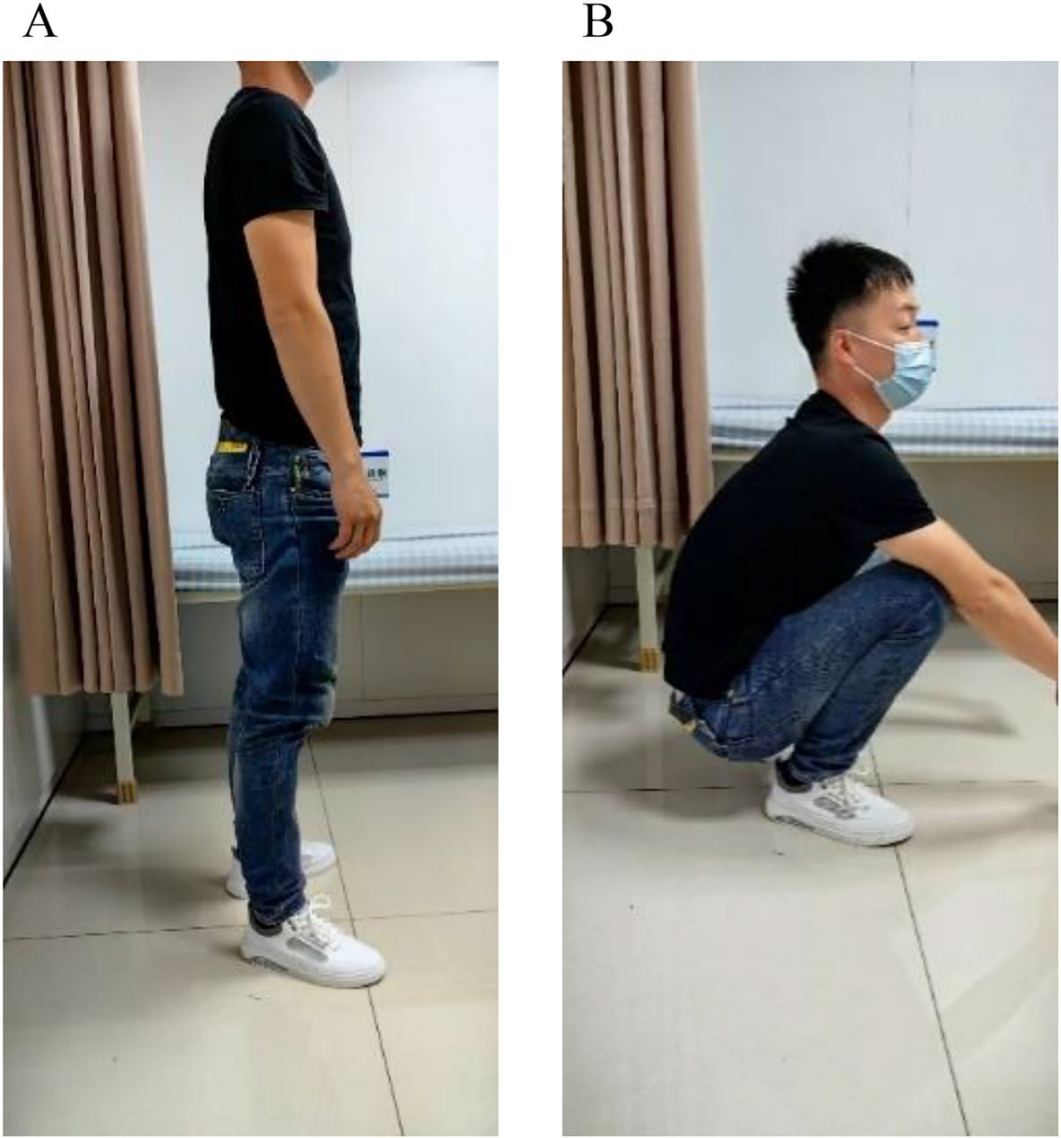
The status of the male patient 12 months after surgery. **A** and **B**: 12 months after surgery, the patient’s gross functional position photos showed good functional recovery of the affected hip, and the hip Harris score was 96.

**Fig. 4:**
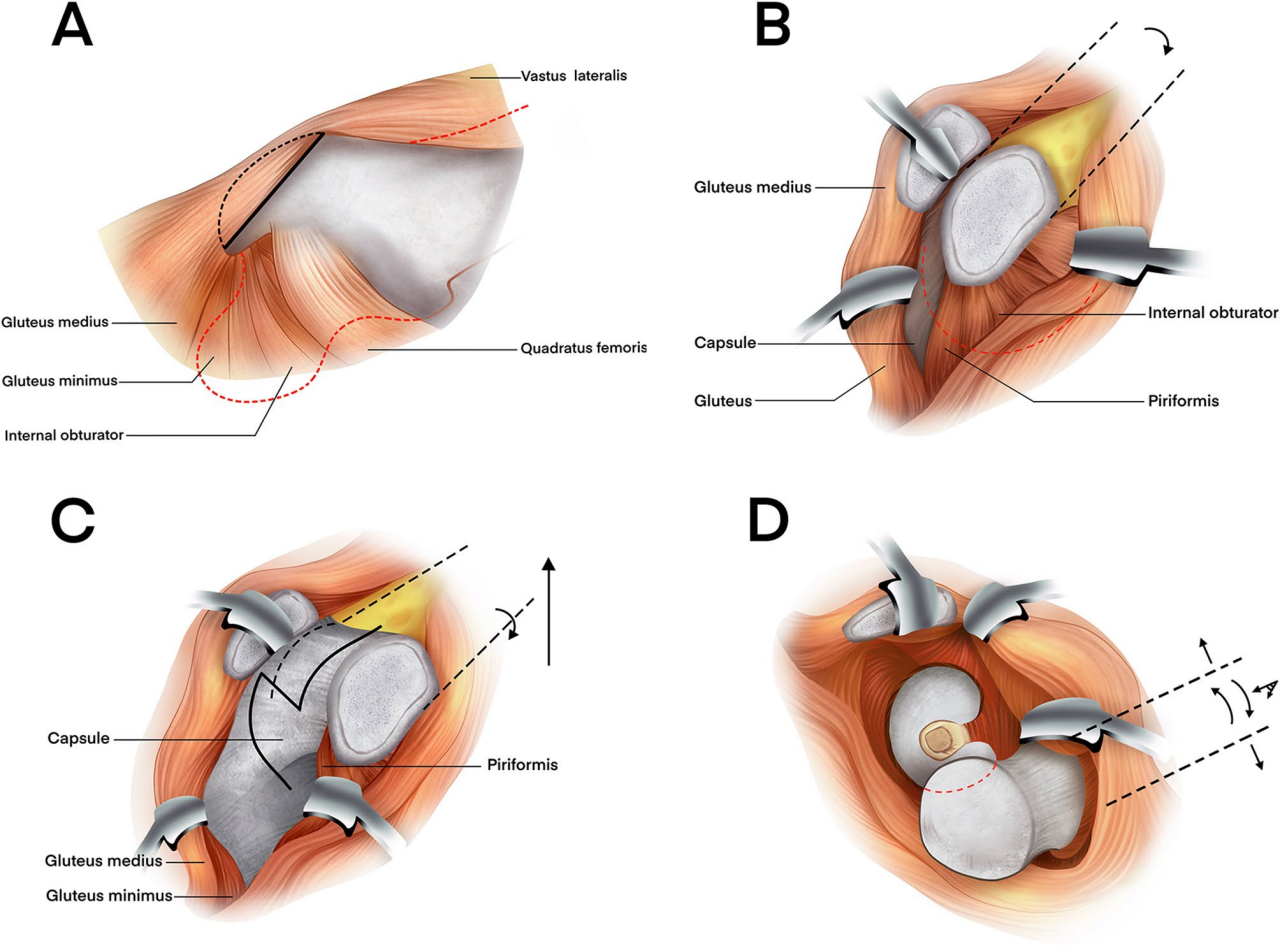
Osteosynthesis of a femoral head fracture using the Ganz approach

General anaesthesia with tracheal intubation was used in all patients. After successful induction, the patient was positioned in the lateral decubitus position with the unaffected side down. The affected hip and lower limb were then disinfected and draped in a sterile fashion.Patients in group A selected Ganz’s approach (Fig. 4), which has been well described in previous literature. The Ganz approach preserves the short external rotators and the medial femoral circumflex artery, offering wide exposure of the hip joint for simultaneous treatment of femoral head and some acetabular fractures, removal of loose fragments, and stable fixation. However, it requires trochanteric flip osteotomy and reduction of anterior column fractures can be challenging. After satisfactory reduction, a 2mm Kirschler wire was used for temporary fixation, and 2–3 double-head compression screws were applied according to the size of the fracture fragment (Weigao, Shandong, China) to expose the posterior fracture fragment, remove the soft tissue on the medial side of the posterior wall fracture fragment, and retain the articular capsule attachment as much as possible. The posterior wall of the acetabular mass fracture could be fixed with a post-reduction posterior wall support with a post-reduction delay screw and a 3.5 mm reconstruction plate. Small posterior wall fractures can be fixed with micro-locking plates and screws (Weigao, Shandong, China), and the hip joint can be easily reduced after the operation. 2–3 7.5 mm hollow lag screws or micro-locking plates (Weigao, Shandong, China) can be used for fixation of the greater trochanter osteotomy (Figs. 1, 2 and 3).

**Fig. 5:**
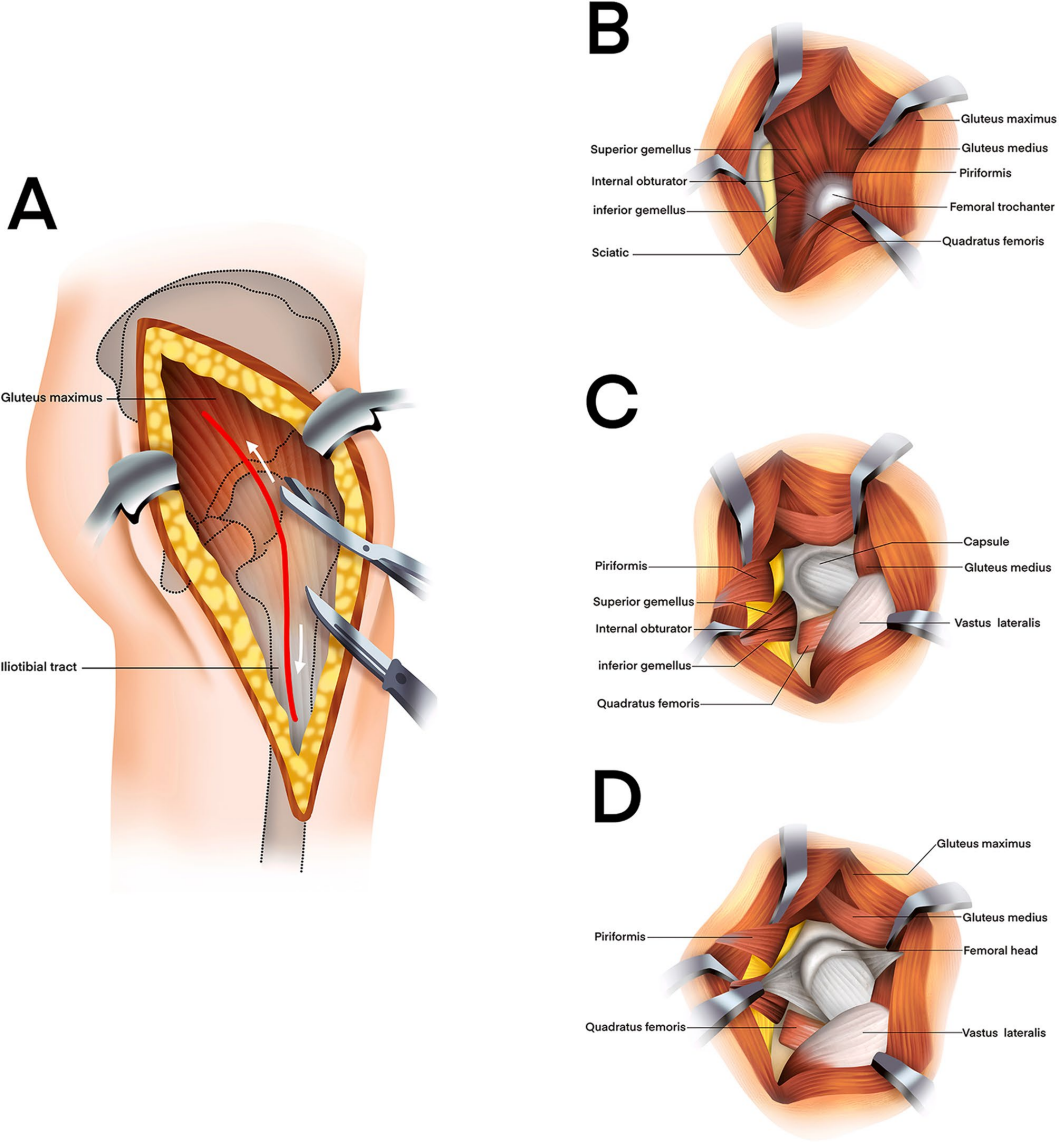
Osteosynthesis of a femoral head fracture using the Kocher-Langenbeck approach Diagram **A** showing the skin and subcutaneous tissue incision, followed by division of the fascia lata laterally. The gluteus maximus is bluntly split along its fiber orientation at the junction of the upper and middle thirds.Diagram **B**–**C** showing that with retraction of the gluteus maximus, the short external rotators (piriformis, obturator internus, and gemellus) are exposed. Internal rotation of the femur (arrow) tenses these muscles, facilitating safe dissection away from the sciatic nerve. The rotators are transected approximately 1 cm medial to their insertion on the greater trochanter, exposing the posterior joint capsule.Diagram **D** showing that a T-shaped capsulotomy is performed. For extended exposure, the insertions of the quadratus femoris and gluteus maximus may be partially released

In group B, a K-L incision was performed from 5 cm below the posterior superior iliac spine, extending laterally and distally through the greater trochanter for 10–15 cm(Fig. 5). After splitting the gluteus maximus and fascia lata, the short external rotators were detached 0.5–1 cm from the greater trochanter and tagged for later repair, taking care to protect the sciatic nerve if injured. The posterior column fracture line was exposed, and soft tissue was cleared from the fracture site. Under direct vision, the femoral head fracture was reduced by gentle traction and manipulation of the limb, ensuring anatomical alignment, and temporarily fixed with Kirschner wires before definitive fixation with plates and screws. During the procedure, the quadratus femoris was preserved to protect the ascending branch of the medial circumflex femoral artery, and the gluteus medius was safeguarded from superior gluteal artery injury.

The K-L approach provides good exposure, facilitates repair of posterior wall acetabular fractures and the joint capsule, and enables reduction and fixation of femoral head fractures. However, this approach does not allow direct visualization of fracture fragments located at the anteroinferior aspect of the femoral head. While it may avoid disrupting the anterior capsular blood supply and thus benefit femoral head perfusion, it can compromise the major posterior blood supply to the femoral head, which may increase the risk of avascular necrosis.

Here is the section on postoperative management and efficacy evaluation. The affected limb after operation was kept in an abductive position of external rotation for 24 h in order to avoid adduction and internal rotation. Broad-spectrum antibiotics were given 30 min preoperatively, broad-spectrum antibiotics were routinely given within 24 h of surgery to prevent infection, rivaroxaban was given as an oral anticoagulant for 2 weeks to prevent thrombosis, indomethacin was used to prevent ectopic ossification, and symptomatic treatment such as detumescence and analgesia was given. Place drainage in the wound for 1–3 days (remove the drainage tube when it is lower than 50 ml/day). Hip joint X-ray and CT scan were performed to check fracture alignment and healing. Patients were encouraged to perform passive hip movement early, and the quadriceps isometrics were performed on the affected side the first day after surgery. Active and passive activities of the affected lower limb were started 1 to 2 weeks after surgery, depending on the intraoperative assessment of the stability of the fixation and its ability to tolerate activity.The function of the affected limb was exercised on crutches 8 to 12 weeks after surgery under non-weight-bearing conditions. The outpatient department regularly reviewed the X-ray film and guided the patient to perform functional exercise. After 12 weeks, the patient was instructed to perform partial weight bearing functional exercises in accordance with the results of the X-ray review to assess the fracture healing situation, and the load weight gradually increased until the full weight bearing. The postoperative regimen was similar in both the Ganz and K-L groups, and patients were regularly followed up by X-ray and CT scans.

Outcome measures Postoperatively, an x-ray of the pelvis and a 3D reconstruction of the CT were performed to understand the fracture reduction. The results were checked monthly for the first 6 months and every 3 months until fracture healing. Fracture healing was defined as the presence of continuous trabecular bone crossing the fracture line on radiographs, no fracture line widening, and absence of local pain or discomfort during functional activities [12]. Femoral head healing and acetabular healing were assessed separately: femoral head healing was evaluated on anteroposterior and lateral hip radiographs and confirmed by CT when necessary, while acetabular healing was evaluated on pelvic radiographs and CT according to Matta imaging criteria. Incidence of fracture healing and FH avascular necrosis was observed. Matta imaging criteria were used to assess the quality of acetabular fracture reduction. The degree of displacement seen on X-ray and CT scans was used to evaluate the method. Displacement 0–1 mm was anatomic reduction, displacement 2–3 mm was satisfactory reduction, and displacement >3 mm was unsatisfactory reduction. The reduction quality of FHF was assessed by Thompson-Epstein clinical and imaging evaluation criteria. The scoring system comprehensively evaluated pain, range of motion, walking and imaging findings, and was divided into four grades: excellent, good, fair and poor. Among them, no pain, no mobility disorder, no claudication and progressive imaging changes of the hip were considered good, mild claudication or mild imaging changes were considered good, mild pain, limited hip mobility (no adduction disorder), moderate claudication or moderate imaging changes were considered OK, once severe pain, significant limited hip mobility (adduction disorder), re-dislocation of the hip, or severe imaging changes were found to be poor. Hip function was assessed by the Harris hip score at the last follow-up. All radiologic assessments, including Matta imaging criteria and Thompson–Epstein clinical and radiological evaluations, were independently reviewed by two senior orthopedic surgeons who were blinded to the surgical approach and clinical outcomes. Any discrepancies between the two reviewers were resolved by consensus discussion. Due to the small sample size and retrospective nature of the study, interobserver reliability (e.g., kappa or ICC) was not formally calculated.

### Statistical analysis

IBM SPSS 27.0 statistical software (IBM, USA) was used for statistical analysis. For measurement data, to determine whether the data were distributed normally, the Shapiro-Wilk test was used. Age, operative time, intra-operative blood loss, fracture healing time, Harris score, and hip motion were normally distributed data, and the variance was homogeneous and was expressed as $\:\stackrel{-}{x}\pm\:s$. Comparisons between the two groups were made using a two independent samples t-test. Thompson-Epstein clinical and imaging scores and Matta imaging scores were the grade data, and Man-Whitney U test was used. Gender and side were counted data. The Fisher exact test was exploited to compare the two groups. *P* < 0.05 was considered to be statistical difference.

## Results

### Demographic data

**Fig. 6:**
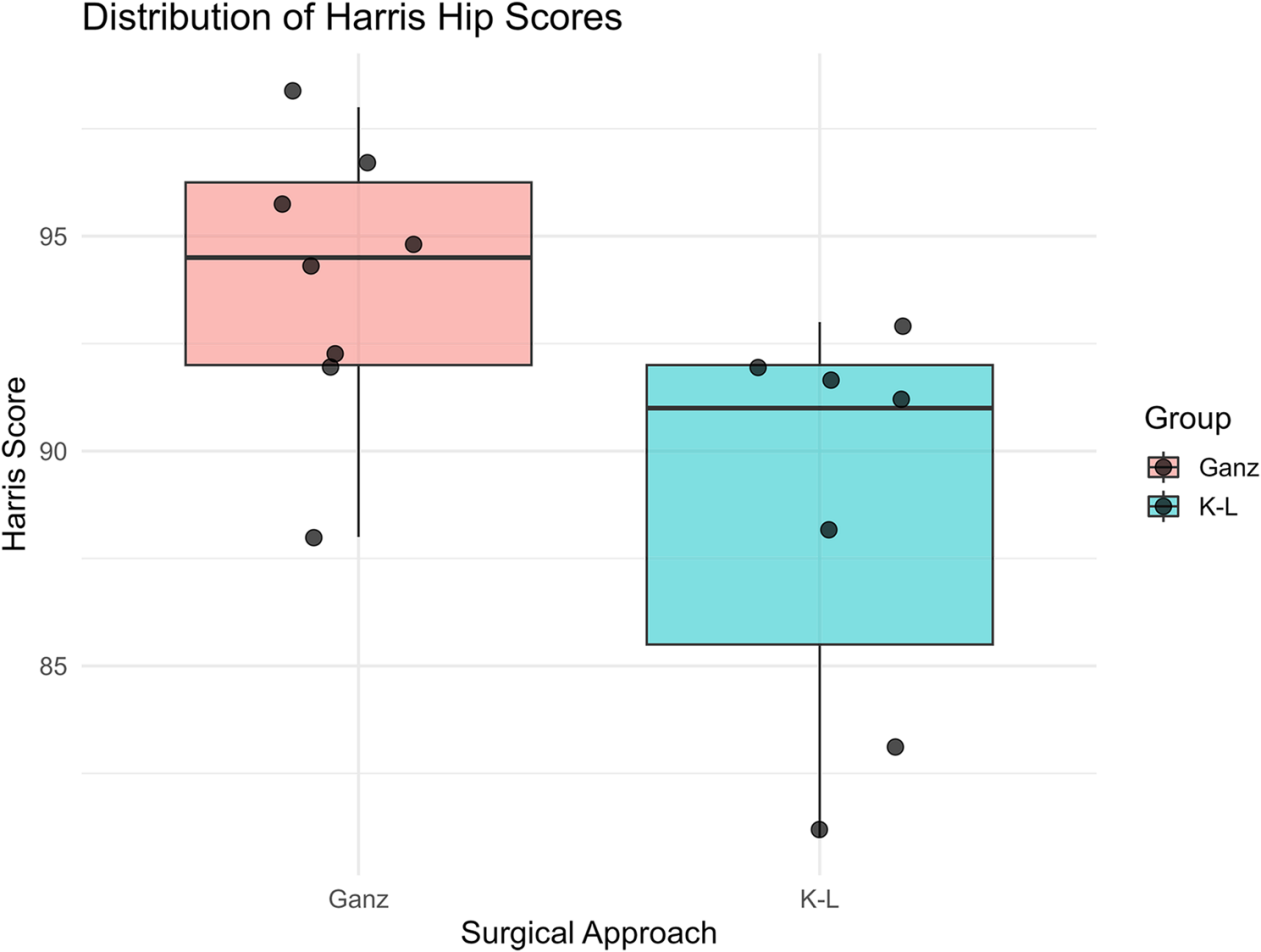
Distribution of Harris Hip Scores Between Ganz and K-L Groups Boxplot with individual data points showing the distribution of Harris hip scores in the Ganz (*n* = 8) and K-L (*n* = 7) groups Y-axis: Harris Hip Score (points); X-axis: Surgical approach (Ganz vs. K–L) Boxes indicate the interquartile range with the median line shown; whiskers represent minimum and maximum values. Mean ± SD values are shown for each group. The Ganz group demonstrated significantly higher postoperative functional scores compared with the K–L group (*P* < 0.05, two-sample t-test)

A total of 15 patients were enrolled from Ningbo No.6 Hospital between March 2019 and April 2022, including 12 males and 3 females, ranging in age from 23 to 66 years, with an average age of 40.5 years. Twelve injuries were due to traffic accidents, two falls from height and one Sport injury. Combined injuries: 1 case of limb fracture, 1 case of spinal injury. All patients were operated by two senior physicians. All patients received 12–23 months of follow-up, averaging 15.8 months. All combined injuries were satisfactorily treated, and the remaining fractures healed within 6 months. No significant difference was found between the two groups in preoperative general data (*P* > 0.05) (Table 1). Hip joint function, assessed by the Harris hip score, was significantly better in the Ganz group compared to the K-L group (*P* < 0.05) (Table 2; Fig. 6). The distribution of Harris hip scores is illustrated in Fig. 6, showing consistently higher functional outcomes in the Ganz group. Range of motion parameters including hip flexion/extension, internal/external rotation, adduction, and abduction were slightly better in the Ganz group, though not statistically significant (*P* > 0.05) (Table 2).

**Table 1:** Comparison of preoperative general data such as gender, side and age between the Gans approach group and the K-L approach group

Group	Cases	Gender (example)		Side (left and right)		age (years)
		Male	female	left	right	
Gans approach group	8	7	1	5	3	37.37 ± 9.05
K-L approach group	7	5	2	3	4	40.28 ± 13.14
test statistics	-	0.608		0.582		0.505
P value	-	0.569		0.619		0.622

**Table 2:** Comparison of Harris score and hip function score 1 year after surgery between Gans approach group and K-L approach group

Group	Cases	Harris score at last follow-up	Hip motion 1 year after surgery					
			Flexion	Extention	Internal rotation	external rotation	adduction	abduction
Gans approach group	8	94 ± 3.25	114.37 ± 21.11	10.37 ± 5.04	37.25 ± 9.13	31.75 ± 6.81	22.50 ± 7.07	32.25 ± 7.20
K-L approach group	7	88.57 ± 4.79	104.285 ± 19.24	11.42 ± 6.37	34.28 ± 6.72	28.57 ± 5.56	20.00 ± 7.09	25.71 ± 8.09
test statistics	-	2.599	0.962	−0.357	0.706	0.979	0.682	1.655
P value	-	0.022	0.354	0.726	0.492	0.484	0.507	0.122

**Table 3:** Comparison of operative time, intraoperative blood loss and fracture reduction quality between the Gans approach group and the K-L approach group

Group	Cases	Operation time (min)	Intraoperative blood loss (ml)	Evaluation of Matta imaging criteria		Thompson-Epstein clinical and imaging scores		Fracture healing time
				Anatomy	Satisfactory	excellent	Good	
Gans approach group	8	132.50 ± 34.12	412.50 ± 164.20	6	2	7	1	13.37 ± 3.5
K-L approach group	7	160 ± 12.72	500 ± 163.29	5	2	2	5	16.28 ± 2.81
test statistics	-	−1.567	−1.032	63		47.500		−1.743
P value	-	0.141	0.321	1.000		0.041		0.105

### Radiological outcomes

Matta imaging scores indicated comparable quality of fracture reduction between the two groups, with no significant difference (*P* > 0.05) (Table 3). The Ganz group showed superior Thompson-Epstein clinical and imaging scores (*P* < 0.05) (Table 3). Fracture healing time was shorter in the Ganz group, but the difference was not statistically significant (*P* > 0.05).

### Complications

In the Ganz approach group, there was one case of preoperative sciatic nerve injury, which improved after three months of mecobalamin treatment, and one case of post-traumatic arthritis without femoral head necrosis. In the K-L approach group, one case of post-traumatic arthritis was also observed. No reoperations occurred in either group.

## Discussion

This study found that the Ganz approach achieved better functional outcomes than the K-L approach for Pipkin type IV femoral head fractures, with significantly higher Thompson–Epstein and Harris scores. Although differences in operative time, blood loss, fracture healing time, and hip range of motion were not statistically significant, the Ganz group showed numerically better outcomes in these parameters. However, given the limited sample size and lack of statistical significance, these findings should be interpreted with caution, and no firm conclusions can be drawn. Larger studies are needed to determine whether these differences represent true effects. Femoral head necrosis was less frequent in the Ganz group, and complication rates were similar, supporting our hypothesis that its superior exposure and reduction quality contribute to improved recovery.

Our study demonstrated that patients undergoing the Ganz surgical approach achieved significantly higher Thompson–Epstein and Harris scores compared to those treated via the K-L approach, indicating better postoperative functional recovery. This finding aligns with previous reports that Ganz’s SHD technique provides almost complete circumferential exposure, facilitating more precise fracture reduction and stable internal fixation, thereby improving functional outcomes [7, 13]. Recent meta-analyses and large case series have also evaluated these surgical approaches. For example, Khalifa et al. (2021) [14] conducted a systematic review and meta-analysis showing that surgical hip dislocation provides reliable exposure and satisfactory outcomes, although the risk of heterotopic ossification should be considered. Similarly, Massè et al. (2015) [10] and Solberg et al. (2009) [15] reported favorable functional results and acceptable complication rates in larger cohorts of patients undergoing Ganz-style approaches for Pipkin fractures. These findings are consistent with our observations that the Ganz approach yields better functional outcomes than the K-L approach, despite the limitations of our small sample size. Numerous case series and reviews have also reported favorable clinical scores following SHD or trochanteric-flip/trochanteric-osteotomy (i.e., Ganz-style flip osteotomy) for Pipkin or complex femoral head fractures [9, 10, 15]. These studies provide strong evidence supporting our proposed mechanism, whereby improved surgical visualization leads to more accurate fracture reduction, ultimately resulting in better functional outcomes. The Ganz approach preserves femoral head perfusion and offers excellent joint visualization for simultaneous management of femoral head and acetabular fractures, but remains technically demanding for anterior column injuries. This study differs somewhat from certain previous reports, which may be attributed to several factors. This may be attributed to variations in case selection (differences in Pipkin fracture type distribution), follow-up duration, surgeon experience, or inconsistencies in rehabilitation protocols. Although operative time, intraoperative blood loss, healing time, and range of motion did not reach statistical significance in our study, all these parameters numerically favored the Ganz approach. This observation is consistent with several previous reports: despite involving additional procedural steps—such as trochanteric flip osteotomy—the excellent surgical exposure provided by the Ganz approach often facilitates faster fracture reduction and avoids the need for auxiliary incisions to improve visualization, thereby maintaining overall surgical efficiency comparable to traditional posterior approaches [16, 17]. Furthermore, multiple reviews and case series have indicated that SHD results in acceptable intraoperative blood loss and healing profiles that are not significantly greater than those associated with conventional posterior approaches [13, 18]. Possible reasons for discrepancies with previous studies include differences in the statistical definitions of “blood loss” and “operative time” (e.g., whether bone osteotomy or wound closure time is included), variations in surgical proficiency across centers performing the same approach, and case complexity—such as the presence of polytrauma or the need for concomitant acetabular fixation. Our relatively small sample size further limits statistical power, making it difficult to detect potentially clinically meaningful trends.

In our study, the incidence of avascular necrosis was lower in the Ganz group compared to the K-L group, with no heterotopic ossification observed in either group during the follow-up. However, given the very small sample size and the relatively short follow-up, this finding cannot be considered conclusive. Heterotopic ossification is a recognized complication following surgical hip dislocation, and its absence in our series should be interpreted with caution. Future multicenter studies with larger cohorts and longer follow-up are needed to more accurately assess the true incidence of this complication. These findings are consistent with the original and subsequent studies on the Ganz approach, which demonstrate that an appropriately performed trochanteric flip osteotomy combined with careful soft tissue handling can provide adequate exposure while preserving the deep branch of the medial femoral circumflex artery (MFCA) blood supply, thereby reducing the risk of AVN [16, 17]. Conversely, some studies have reported that surgical SHD may be associated with a higher risk of heterotopic ossification or local complications—such as those documented in multicenter complication registries—although the overall avascular necrosisrates remain low [14, 19]. Potential reasons for discrepancies between our findings and some previous reports include the extent of vascular injury at the time of trauma, the timing of fracture reduction, individual patient factors (e.g., smoking status, comorbidities), and the duration of follow-up, all of which substantially influence avascular necrosis occurrence.

We believe that the advantages of the Ganz approach primarily stem from its ability to provide more comprehensive visualization and a more direct pathway for reduction, which facilitates the simultaneous management of osteochondral fragments, intra-articular loose bodies, and posterior acetabular wall injuries. This, in turn, improves the quality of reduction and may enhance functional outcomes. Multiple anatomical and technical studies have emphasized that trochanteric flip osteotomy, combined with exposure through the correct fascial and tendinous intervals, enables full circumferential visualization of both the acetabulum and femoral head. Importantly, this approach does not compromise the main trunk of the MFCA [6, 7]. These findings are consistent with our surgical experience. Previous comparative studies and systematic reviews have also corroborated the logical sequence in which better visualization leads to superior radiological reduction scores, which in turn result in improved functional outcomes.

This study has several limitations. Firstly, it was a retrospective, single-center case series with a very small sample size of 15 patients, which reduces statistical power, increases the risk of selection bias, and limits the generalizability of the findings. The rarity of Pipkin type IV fractures and the strict inclusion and exclusion criteria contributed to the limited sample size. Because of the small cohort, we were also unable to perform multivariable regression analysis to adjust for potential confounders such as sex, bone mineral density, fracture characteristics, and surgical approach. This may have introduced residual confounding, and future multicenter studies with larger sample sizes should include multivariable adjustment to validate our findings. In addition, the relatively short follow-up period (12–23 months) may not be sufficient to fully assess late complications such as avascular necrosis or post-traumatic arthritis. Therefore, our findings should be interpreted with caution and regarded as supportive of prior research rather than definitive. Future multicenter studies with larger cohorts and longer follow-up (≥ 3–4 years) are warranted to confirm these observations.Secondly, although all surgeries were performed by experienced senior surgeons to maintain consistency, the results may not be generalizable to other institutions with different surgical expertise. Future multicenter, prospective studies are needed to minimize bias and improve the robustness of the evidence. Thirdly, some patients in our cohort sustained associated injuries, including one case of spinal injury and one case of limb fracture. Although these combined injuries were treated successfully and healed within 6 months, their potential impact on hip functional recovery could not be adequately evaluated in our analysis. The very small number of such cases precluded subgroup comparisons, and future multicenter studies with larger cohorts are needed to further clarify the influence of concomitant injuries on outcomes. All surgeries were performed by two experienced surgeons, which may limit the applicability of the results to other surgeons or centers. The follow-up period in this study was relatively short (12–23 months), which is insufficient to fully evaluate late complications such as avascular necrosis or post-traumatic arthritis, as these conditions may take several years to become clinically apparent. Therefore, our findings regarding complication rates should be interpreted with caution. Future studies with extended follow-up periods are necessary to provide a more comprehensive assessment of long-term outcomes.Important patient-related factors, including rehabilitation protocols, comorbidities, smoking status, nursing care, and nutrition, were not systematically controlled in this study, which may have introduced confounding effects. Future studies should apply standardized rehabilitation protocols and collect detailed patient-related information to minimize bias and improve reliability. Furthermore, although efforts were made to maintain consistency between surgeons, a fully standardized protocol for implant selection and intraoperative decision-making was not applied. This may have introduced variability, and future prospective studies with unified surgical protocols are needed to reduce heterogeneity. Finally, although radiologic evaluations were independently performed by two experienced surgeons, interobserver reliability was not formally assessed, which may introduce subjective bias in imaging-based outcome classification. We have noted these limitations in the manuscript and suggest that future studies use multicenter, prospective designs with larger samples and longer follow-up to confirm our findings.

## Conclusion

The Ganz approach may be an ideal approach for the treatment of Pipkin type IV fracture. Compared with the traditional K-L approach, the Ganz approach offers better visual field exposure, better reduction quality of FH, and better postoperative functional recovery. However, given the small sample size of this study, larger multicenter studies with longer follow-up (≥ 3–4 years) are necessary to confirm these results.

## Data Availability

The datasets used and/or analysed during the current study available from the corresponding author on reasonable request.
