# Correction: Comparison of clinical efficacy of Ganz approach and K-L approach in treatment of Pipkin type IV femoral head fracture

**DOI:** 10.1186/s12893-026-03520-z

**Published:** 2026-02-24

**Authors:** Jun Zhang, Songqi Chai, Li Dai, Yunqiang Zhuang

**Affiliations:** 1 Department of Trauma Orthopedic Center, Ningbo No.6 Hospital, Ningbo, Zhejiang China; 2 Ningbo Clinical Research Center for Orthopedics, Sports Medicine & Rehabilitation, No.1059 Zhongshan East Road, yinzhou District, Ningbo, 315040 China; 3 Health Science Center, Ningbo University, Ningbo, 315211 China

**Correction to: BMC Surg 25, 587 (2025)**

**https://doi.org/10.1186/s12893-025-03301-0**

In this article [1], The Figs. 1 and 3 appeared incorrectly and have now been corrected in the original publication. For completeness and transparency, the old incorrect and correct versions are displayed below. The original article has been corrected.

Incorrect Fig. 1:

**Fig. 1:**
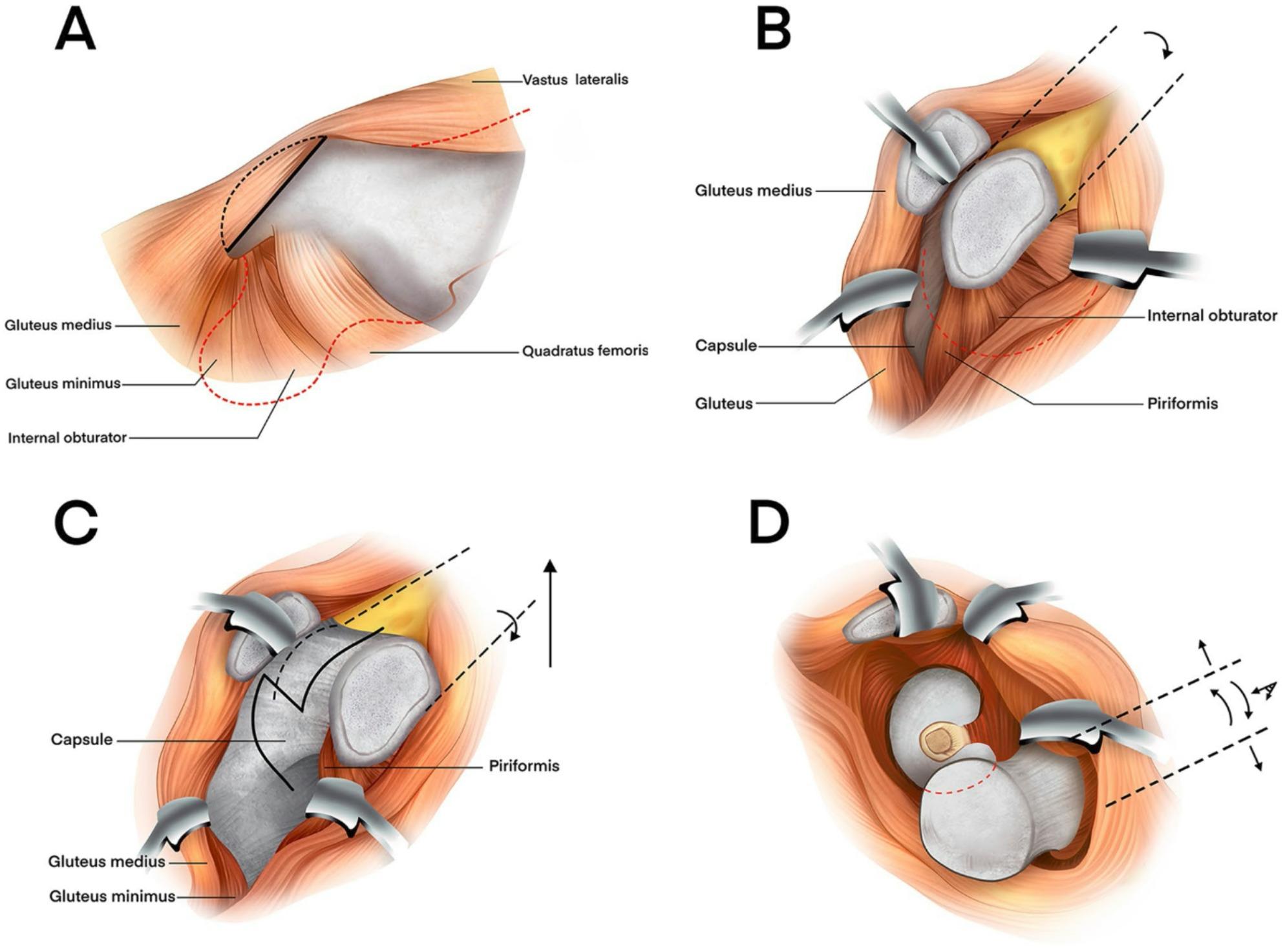
A 32-year-old male patient with a left hip injury caused by a traffic injury. Diagnosis: Fracture of the left femoral head (Pipkin type IV), fracture of the left posterior wall of the acetabulum. Greater trochanter osteotomy was performed 7 days after injury using Ganz approach under general anesthesia. The left acetabular fracture and left femoral head fracture were treated with open reduction and internal fixation. The acetabular fracture was fixed with plates and screws, the femoral head fracture was fixed with counterburied screws, and the greater trochanter was fixed with screws and plates. A-C: Preoperative CT scan showed a fracture of the left femoral head (Pipkin type IV) with significant displacement of the fracture end and a fracture of the posterior lateral wall of the acetabulum

Incorrect Fig. 3:

**Fig. 2:**
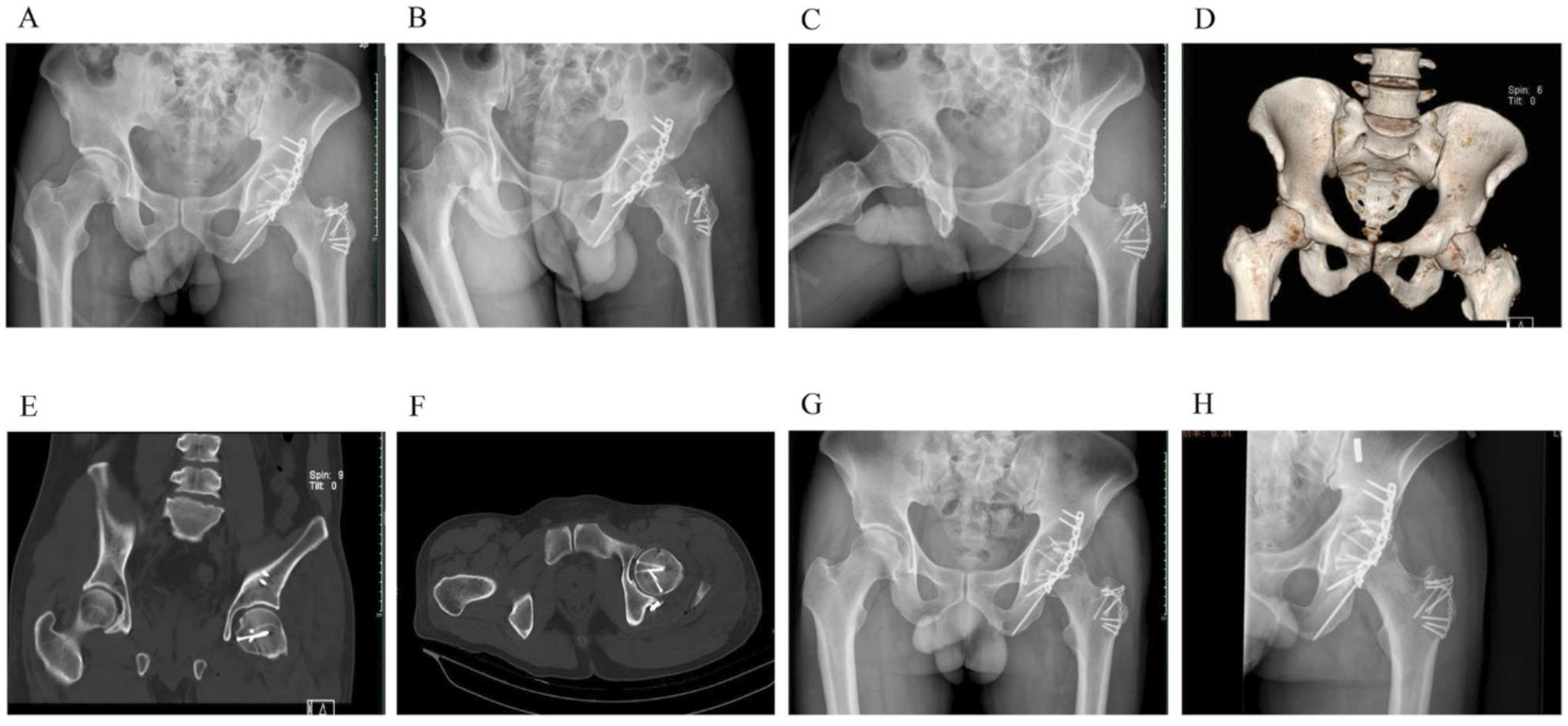
The status of the male patient 12 months after surgery. A and B: 12 months after surgery, the patient's gross functional position photos showed good functional recovery of the affected hip, and the hip Harris score was 96

Correct Fig. 1:

**Fig. 3:**
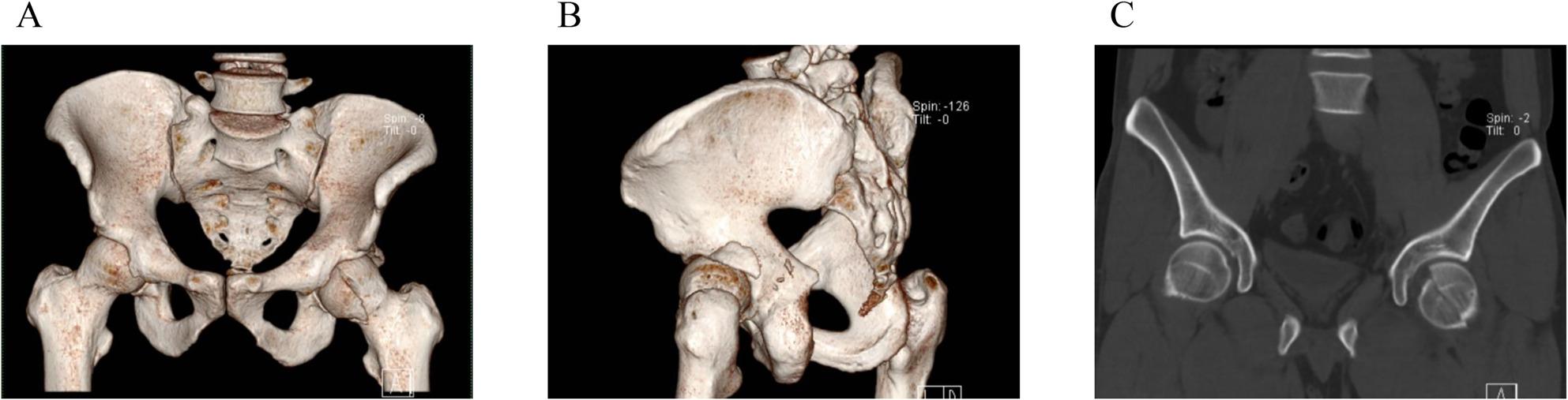
A 32-year-old male patient with a left hip injury caused by a traffic injury. Diagnosis: Fracture of the left femoral head (Pipkin type IV), fracture of the left posterior wall of the acetabulum. Greater trochanter osteotomy was performed 7 days after injury using Ganz approach under general anesthesia. The left acetabular fracture and left femoral head fracture were treated with open reduction and internal fixation. The acetabular fracture was fixed with plates and screws, the femoral head fracture was fixed with counterburied screws, and the greater trochanter was fixed with screws and plates. A-C: Preoperative CT scan showed a fracture of the left femoral head (Pipkin type IV) with significant displacement of the fracture end and a fracture of the posterior lateral wall of the acetabulum

Correct Fig. 3:

**Fig. 4:**
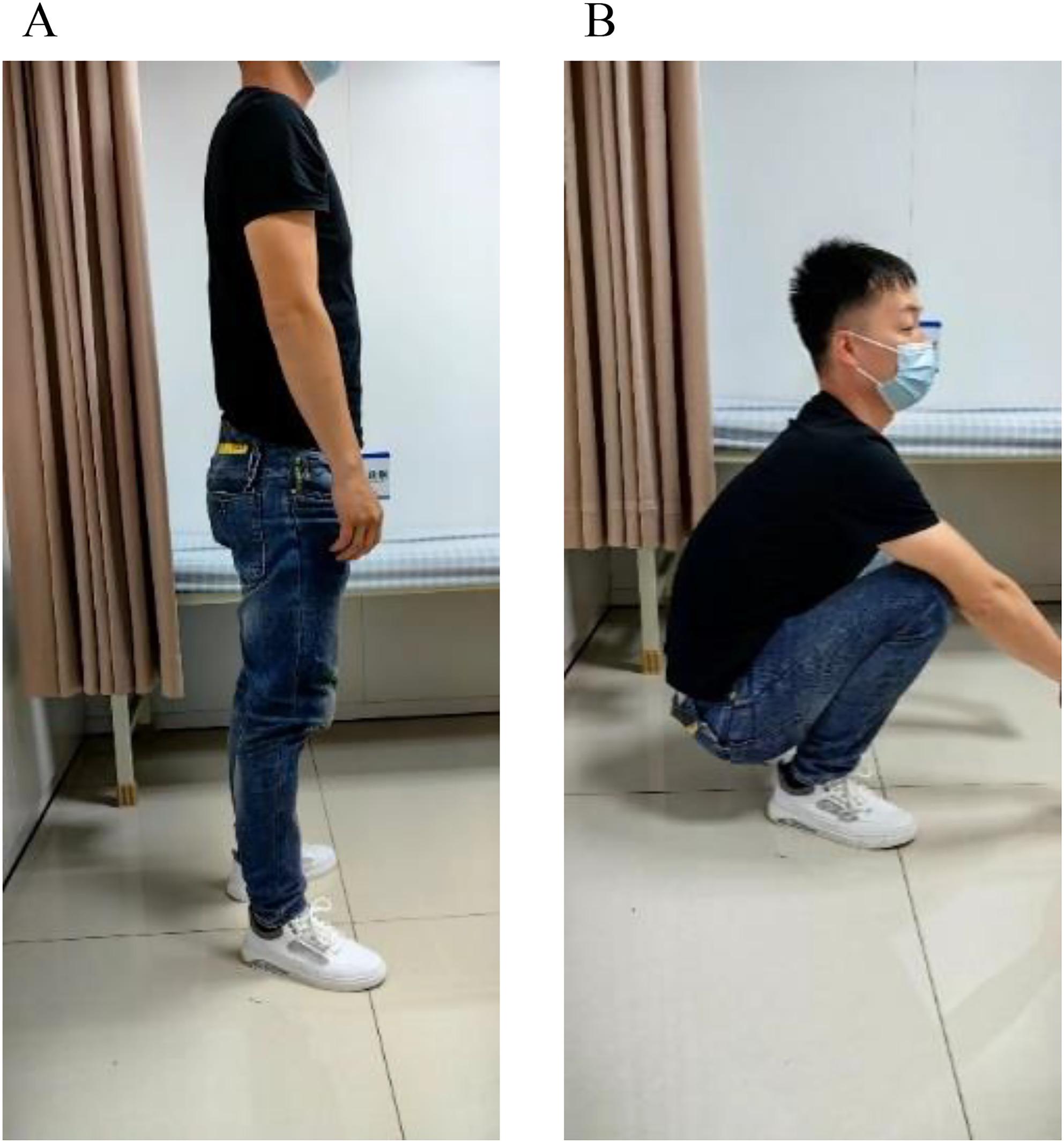
The status of the male patient 12 months after surgery. A and B: 12 months after surgery, the patient's gross functional position photos showed good functional recovery of the affected hip, and the hip Harris score was 96
